# Minimal Residual Disease in Acute Myeloid Leukemia: Still a Work in Progress?

**DOI:** 10.3390/jcm6060057

**Published:** 2017-06-03

**Authors:** Federico Mosna, Debora Capelli, Michele Gottardi

**Affiliations:** 1 Hematology and Bone Marrow Transplantation Unit, Ospedale Centrale “San Maurizio”, Azienda Sanitaria dell’Alto Adige, via L. Bohler 5, 39100 Bolzano, Italy; 2 Hematology, Ospedali Riuniti di Ancona, 60121 Ancona, Italy; debora.capelli70@gmail.com; 3 Hematology, Ospedale “Ca’ Foncello”, AULSS 2, 31100 Treviso, Italy; michele.gottardi@aulss2.veneto.it

**Keywords:** acute myeloid leukemia, minimal residual disease, allogeneic transplantation, leukemia-initiating cells, leukemia stem cells, next generation sequencing, multiparameter flow cytometry

## Abstract

Minimal residual disease evaluation refers to a series of molecular and immunophenotypical techniques aimed at detecting submicroscopic disease after therapy. As such, its application in acute myeloid leukemia has greatly increased our ability to quantify treatment response, and to determine the chemosensitivity of the disease, as the final product of the drug schedule, dose intensity, biodistribution, and the pharmakogenetic profile of the patient. There is now consistent evidence for the prognostic power of minimal residual disease evaluation in acute myeloid leukemia, which is complementary to the baseline prognostic assessment of the disease. The focus for its use is therefore shifting to individualize treatment based on a deeper evaluation of chemosensitivity and residual tumor burden. In this review, we will summarize the results of the major clinical studies evaluating minimal residual disease in acute myeloid leukemia in adults in recent years and address the technical and practical issues still hampering the spread of these techniques outside controlled clinical trials. We will also briefly speculate on future developments and offer our point of view, and a word of caution, on the present use of minimal residual disease measurements in “real-life” practice. Still, as final standardization and diffusion of the methods are sorted out, we believe that minimal residual disease will soon become the new standard for evaluating response in the treatment of acute myeloid leukemia.

## 1. Introduction

Despite profound advancements in the therapy of Acute Myeloid Leukemia (AML), a cure is still achieved, at the state of the art, in no more than 30–40% of adult patients [1,2,3], as the significant results achieved with dose-intense chemotherapy and the use of allogeneic Hematopoietic Stem Cell Transplantation (allo-HSCT) are limited by the toxicity of these treatments. Because of the high heterogeneity of AML, it is fundamental to assess risk factors associated with treatment failure, with the goal to individually design therapy to maximize the chances of a cure without overtreatment. Baseline assessment at diagnosis includes disease-related assessment, such as laboratory, cytogenetic, molecular findings, and the development of AML secondary to prior hematological disorders or chemotherapy, as well as patient-related factors such as age, comorbidities, and performance status [2,3,4]. Also, every major advancement in the survival of AML patients has come through the achievement of greater control over a relentless, aggressive disease. Until recent times, the quality of response to treatment has been evaluated on a morphological and cytogenetic basis only, each characterized by limited sensitivity (approximately 1 × 10^−2^), and defined as “complete remission” (CR). As rapidly advancing technologies with much higher sensitivity have enabled us to better evaluate the extent of such remission, it has become clear how a better assessment of the quality of response to therapy beyond CR could provide a better prognostic evaluation of individual patients, and, possibly, determine better clinical decisions. “Minimal Residual Disease” (MRD) evaluation refers to every technique capable of measuring the submicroscopic persistence of leukemia.

In this review, we will address the state of the art of MRD detection in AML in adults (excluding acute promyelocytic leukemia), from a clinical point of view. After more than a decade of research, MRD techniques appear finally ready to substitute morphological assessment of response in both experimental trials and the general clinical praxis [5,6,7].

## 2. Has MRD Evaluation Superseded Baseline Risk Assessment and Disease Staging?

The current prognostic assessment of AML patients relies on a variety of factors reviewed in detail elsewhere [2,3,4]. Briefly, upfront cytogenetic analysis reveals a favorable, intermediate and adverse karyotype in approximately 15–20%, 60–70% and 15–20% of the cases, respectively. This is particularly clear in younger (i.e., <60–65 years) AML patients treated with curative intent. Patients falling into these three categories can expect an approximate 65–70%, 40% and less of 5–10% likelihood of cure, respectively. Karyotype is still considered the most powerful independent predictor of CR achievement, relapse risk and overall survival (OS) [4], with relevant repercussion on treatment approach [2,3]. The general consensus, and a recent meta-analysis [2,3,8,9,10,11,12], recommend the use of allo-HSCT as part of first-line therapy only when the risk of relapse exceeds 35–40% [8,9], such as in cytogenetically adverse-risk AML patients. On the other hand, favorable-risk karyotype AML patients, e.g., Core Binding Factor (CBF) AML patients and normal-karyotype FLT3-wild-type (wtFLT3) NPM1-mutated (mNPM1) patients, are usually not considered candidates for allo-HSCT in first complete remission (CR1). In between, the highly heterogeneous group of intermediate-risk AML has currently no real standard of treatment, as relapse rates in this group vary between 30% and 50% [1,2,3,8,9], and, given a treatment-related mortality (TRM) from allo-HSCT still approaching 15–25% [8,9,10,12], it is highly debated whether to perform allo-HSCT in CR1 or to wait until relapse [2,3]. Improvement in our knowledge of gene mutations in AML has further complicated the baseline prognostic assessment of these patients, that should now include FLT3, NPM1, CEBPA, as well as RUNX1, DNMT3A, IDH1, IDH2, TET2, ASXL1 and TP53 [2,3]. Despite this, with few exceptions, it is still largely unknown how mutations in these genes interact with each other, and how they should direct therapy. Even though baseline characteristics have been associated by several studies to the likelihood of achieving the disappearance of MRD in CR1, most studies have consistently recognized MRD evaluation as an independent prognostic factor for relapse and survival in multivariate analyses [7]. Thus, rather than substituting baseline risk assessment, MRD evaluation could help refine individual prognosis by adding a unique post-hoc evaluation of the chemosensitivity of leukemic blasts and of the residual disease burden after therapy. To do this, different techniques have been tested, with relevant, yet sometimes conflicting, results.

## 3. Molecular Biology Techniques

Molecular techniques rely on the presence of a molecular transcript (RNA) or aberrant gene fusion (DNA) specifically related to AML and conserved throughout disease history. As such, DNMT3A mutation, RUNX1/RUNX1T1, CBFB/MYH11 have all been proven to be early markers of pre-leukemic clones [13,14,15,16,17], while c-KIT, FLT3-ITD/TKD and NPM1 mutations have all been identified as later events in leukemogenesis. This has consequences in the interpretation of persisting molecular MRD after therapy as a predictor of eventual clinical relapse. Furthermore, only approximately 50–60% of AML are marked by the presence of recurrent genetic abnormalities [6,18]. Another approach to MRD by molecular assays would therefore evaluate the persistent overexpression by leukemic cells of genes normally expressed at a much lower rate, such as WT1 [19]. Table 1 summarizes the major molecular MRD markers currently used in AML.

### 3.1. RUNX1/RUNX1T1 and CBFB/MYH11

CBF AML is determined by the disruption of CBF signaling by recurrent chromosomal translocations, either t(8;21)(q22;q22), corresponding to the formation of the RUNX1/RUNX1T1 fusion gene (formerly known as AML1/ETO), or inv(16)(p13q22)/t(16;16)(p13q22), producing the aberrant CBFB/MYH11. Both markers have been used to assess MRD in a variety of retrospective trials, all consistently identifying the persistence or reappearance of these transcripts (>1 × 10^−2^) as predictive of clinical relapse [20,21,22,23]. Either post-induction or end of consolidation appeared as trustworthy time-points for MRD assessment [24,25,26,27]. Moreover, even if one study identified the absolute baseline transcript levels as predictive of poorer prognosis [24], most other studies identified the quantitative trends of RUNX1/RUNX1T1 and CBFB/MYH11 by RT-qPCR, rather than the absolute values in single time-points, as best predictive of relapse [20,24,27,28,29]. For instance, Stentoft et al. identified a 2-log reduction of RUNX1/RUNX1T1 after induction as the most indicative cut-off for response [20], as did Leroy et al. [28], although with a slightly different cut-off (3-log reduction) [28,29]. In all studies, a rising trend in MRD levels during follow-up reliably predicted relapse [6].

A more recent analysis, then, on 278 CBF AML, demonstrated that the persistence or reappearance of RUNX1/RUNX1T1 above 500/10^4^ ABL copies in BM, or above 100/10^4^ ABL copies in PB, after consolidation therapy or during follow-up, were associated with certain hematological relapse [27]. The same was true for CBFB/MYH11 above 50/10^4^ ABL copies in BM, and >10/10^4^ ABL copies in PB, at the same time-points. In the same study, a 3-log reduction cut-off post-induction proved to be the strongest predictor for relapse in the multivariate analysis (3-year CIF 4% vs. 30%) [27]. Despite this, no difference in OS was shown, probably because of a relatively good rescue rate by second-line therapy including allo-HSCT [27].

Interestingly, when Gemtuzumab Ozogamicin (GO), an anti-CD33 immunotoxin, was used in some of these trials as a randomized addition to chemotherapy, “GO+chemotherapy” obtained higher rate of MRD negativity as compared to chemotherapy alone in all cases [27,30,31,32]. Marked molecular responses in CBF AML patients treated with GO and intensive chemotherapy have also been independently reported, with 38 out of 43 patients (88.4%) achieving >3-log reduction of transcript level by the end of the second consolidation cycle [33]. In most studies MRD proved the most powerful independent predictor at the multivariate analysis [20,27,28,29].

After treatment, RUNX1/RUNX1T1 and CBFBMYH11 can remain measurable by RT-qPCR also in patients persisting long-term in CR [27]. Possible biological explanations for that involve the persistence of RUNX1/RUNX1T1 in pre-leukemic clones [13,17], as well as the development of an immunosurveillance effective in preventing disease reoccurrence by the autologous immune system or by Graft-vs-Leukemia effects after allo-HSCT. Both markers have also been reported in healthy individuals [34]. The possible persistence of the CBF transcripts in cured patients stresses out the importance of evaluating their trend over serial time-points, especially in the case of allo-HSCT. Postremission MRD monitoring in time intervals of 3 months is recommended [24,27], and may be also conducted using PB instead of BM, although with a 1-log lower sensitivity [20,28]. Interestingly, a difference in relapse kinetics has been shown between RUNX1/RUNX1T1 and CBFB/MYH11 AML, with CBFB/MYH11 being slower to clinically relapse after molecular MRD reappearance [20].

### 3.2. NPM1

Mutations in the nucleophosmin-1 gene (NPM1) are detected in approximately 60% of patients with cytogenetically normal AML, and in 30% AML overall [35,36]. More than 50 different mutations in the exon 12 of the NPM1 gene have been described, but three mutation types (A, B, and D) account for 95% of all cases [36]. An interesting model using subclonal analysis by NGS has been set that describes NPM1 mutations as late leukemogenic events occurring in epigenetically altered, DNMT3A-mutated hematopoietic clones [13,16,37]. In the absence of FLT3-ITD, NPM1 mutation confers a relatively more favourable prognosis to AML [2,3,35,38]. Moreover, due to their homogeneous mutation pattern and their constant presence at relapse [37,39], NPM1 mutations have been used as MRD markers. Yet, the best source to analyze (PB or BM), the best time-points and best thresholds for NPM1 are yet to be defined. For instance, Balsat et al. analyzed molecular NPM1 MRD, detected by RT-qPCR, in 152 NPM1-mutated AML patients [40]. Multivariate analysis showed higher 3-year CIR (*p* < 0.001) and shorter OS (HR: 5.1; *p* < 0.001) in patients with an NPM1 reduction after induction <4 log. Similar data were observed in FLT3-ITD+ patients (OS, HR: 2.65, *p* = 0.03), and in patients with abnormal karyotype (OS, HR: 10.75, *p* < 0.001). In FLT3-ITD+ patients, multivariate analysis identified only age, WBC count and 4 log NPM1 PB reduction, but not FLT3-ITD allelic ratio, as predictors of OS. Interestingly, in those 71 patients where allo-HSCT was performed because of FLT3-ITD or adverse cytogenetics, it determined a survival benefit in patients with a post-induction MRD clearance <4 log (HR: 0.25; *p* = 0.047 for OS and Disease-free Survival (DFS)), but not in those with a clearance >4 log (HR: 2.11; *p* = 0.261 for OS, HR: 1.62, *p* = 0.419 for DFS) [40].

In another large study on 346 patients [39], though, obtaining a significant reduction of mNPM1 levels early after induction was not equally important; conversely, the authors identified the absolute mNPM1 level in PB after two cycles of chemotherapy as most predictive for survival (3-year risk of relapse 82% vs. 30%, HR: 4.80, *p* < 0.001; OS 24% vs. 75%, HR: 4.38, *p* < 0.001). Interestingly, MRD positivity after two cycles was the only independent prognostic factor for death in the multivariate analysis (HR: 4.84; *p* < 0.001). The discrepancy between these two studies may be due to differences in the method of analysis and patient series.

In a third study on 252 mNPM1 AML [41,42], the persistence of high levels of NPM1 mutations were significantly correlated with survival at each of four time-points of monitoring (18–60 days, 61–120 days, 121–365 days, >365 days). In multivariate analysis, the level of residual NPM1 was the most relevant factor affecting Event-free Survival (EFS), also in the subgroup of patients undergoing allo-HSCT [41]. Patients after first-line therapy who did not achieve a 3-log clearance of mNPM1 level at early checkpoint (18–60 days) did not show a significantly higher relapse rate (HR: 0.02, *p* = 0.097), as in the study by Ivey et al. [39] and in contrast with the results by Balsat et al. [40]. The kinetics of relapse showed that sampling every 2 months would allow early prediction of 50% of all relapses.

A fourth study [43] also demonstrated mNPM1 levels as highly predictive of relapse at two late checkpoints, after double induction therapy and after consolidation therapy. NPM1 persistence (>10^−4^) impacted on 4-year OS (90% vs. 51%, HR: 1.67, *p* < 0.001) and 4-year CIR (6.5% vs. 53%, HR: 2.24, *p* < 0.001). All 36 patients with more than 200 mNPM1/10^4^ ABL copies in serial post-treatment assessment relapsed in a median of 2 months. mNPM1 transcript levels in BM and PB correlated well (*r* = 0.89; *p* < 0.001).

Immunotherapies, such as GO, have been proposed to eradicate MRD in patients in CR, alternatively to allo-HSCT. However, more studies are needed to properly address this issue. The addition of GO to chemotherapy, although increasing the rate of mNPM1 MRD-negativity (39% vs. 7%, *p* = 0.006 after induction; 91% vs. 61%, *p* = 0.028 thereafter), did not translate into better OS in a recent study [44], nor influenced MRD levels as measured by WT1. Interestingly, in this study mNPM1 MRD persistence also did not affect OS [44].

### 3.3. WT1

As only 50–60% of all AML are characterized by recurrent chromosomal translocations and gene mutations [6,18], attention has been pointed out to genes overly expressed by leukemia cells, such as Wilms’ tumor gene (WT1), as an alternative way to measure MRD [19,45]. Approximately 85–90% of AML overexpress WT1 at diagnosis [18]. Several studies have retrospectively confirmed the role of WT1 as a MRD marker: a first one [46] analyzed 71 AML post-induction with a significant correlation between PB WT1 (>20/10^4^ ABL copies) and the incidence of relapse (100% CIR in WT1+ patients, median time 7 months, vs. 43.7% in WT1− patients, median time 12 months). The conversion of WT1 from negative to positive during follow-up anticipated relapse by mean 2.4 months [46], and was more significant when considered as a trend along serial time-points. In another study by the same group, a significant increase of CIR in patients with WT1 reduction <2 log post-induction (75% vs. 40%; HR: 0.54, *p* = 0.004) or a BM WT1 >250 × 10^−4^ and PB WT1 >50 × 10^−4^ post-consolidation was demonstrated (67% vs. 42%; HR: 0.23, *p* = 0.004) [47], with good concordance between paired BM and PB samples. RT-qPCR positivity of WT1 in PB (cut-off > 0.5% after induction) was associated with a higher risk of 2-year CIR (74% vs. 38%, *p* = 0.00084) and a shorter OS (HR: 3.23, *p* = 0.0007) also in a later series of 183 AML patients [44], thus confirming the possibility to use PB as well as BM in patients in follow-up.

Finally, WT1 reoccurrence predicts morphological relapse also in the setting of allo-HSCT: Candoni et al. [48] analyzed WT1 expression after allo-HSCT in 38 AML, demonstrating a concordance between BM WT1, chimerism and molecular MRD, with a CIR of 100% in patients with BM WT1 levels >70 × 10^−4^; Pozzi et al. analyzed BM WT1 in 122 AML patients before and after allo-HSCT, demonstrating higher CIR (HR: 2.3, *p* = 0.002) in patients with BM WT1 >100/10^4^ ABL copies. They also scheduled pre-emptive treatment of molecular relapse with DLI, obtaining a survival advantage in treated patients (5-year OS 44% vs. 14% in a paired control group; *p* = 0.004) [49].

MRD evaluation by WT1 unfortunately presents some pitfalls: 10–15% of AML do not overexpress this marker, and WT1 sensitivity seem to be lower than other PCR methods, ranging from 1:500 to 1:2000 respectively in BM and PB [50]. Discrimination of residual disease from background expression, and therefore specificity of the assay, can also be problematic: to increase sensitivity and specificity, a novel technique has been proposed based on the concomitant identification of a panel of several overexpressed genes in AML, including WT1 [51].

### 3.4. Other Molecular Markers

(1) FLT3-ITD mutations are present in 25–30% of all AML patients and characterized by high prognostic value [2,3]. Despite this, they require patient-specific primers/probes for PCR and are highly inconstant in relapse [6]. Their use as a MRD marker is at present both unpractical and unreliable.

(2) DNMT3A and IDH1/2 mutations were identified in approximately 10–15% of all AML patients by NGS techniques [52]. They often represent an early event disrupting epigenetic regulation in HSC and leading to pre-leukemic clonal expansion [13,14,15,16]. In a recent study, mutations in DNMT3A, TET2, and IDH2 where concomitantly analyzed in NPM1-mutated patients [39]. Differently from mNPM1, mutations of these genes were detected at low levels (>1 × 10^−4^) also in most remission samples. Frequencies of mNPM1, FLT3-ITD and mutated DNMT3A and IDH1/2 were also analyzed together in another study [53]. Among 24 patients with DNMT3A R882H at diagnosis who received chemotherapy and no allo-HSCT, all 24 showed high-level persistence of the mutation; of these, 16 (67%) remained in CR1 at a median follow-up of 38 months (range: 27–56). IDH1 R132H was detected during follow-up in 1 of 11 patients (9%) at a 1 × 10^−2^ level. IDH2 R140Q, finally, was detected in 8 of 18 patients (44%) at a median 2 × 10^−2^ level, with 5 of the 8 patients (63%) remaining in the first complete remission at 36 months (range: 28–65). Interestingly, when allo-HSCT was performed, it led to the elimination of the DNMT3A mutant clone in 8 of 9 patients (89%) [53]. A third study also showed the persistence of DNMT3A mutations in follow-up samples from patients in long-term remission, suggesting that their elimination may not be essential for cure [54]. IDH1/2 mutations, though, seem different: in a study performed on 31 NPM1-mutated AML patients that analyzed together DNMT3A and IDH1/2 mutations, the latter proved highly correlated with NPM1 (*r* = 0.6818, *p* < 0.0001), and highly predictive for clinical relapse [55]. This is particularly interesting when one considers the existence of specific drugs targeting IDH2 [56,57]. Conversely, also in this study DNMT3A mutations persisted in 40% of patients achieving CR [55]. 

(3) Mutations in the RUNX1 gene define a recently introduced WHO entity with dismal prognosis [58]. Unfortunately, its identification needs a patient-specific customized PCR, due to the lack of a mutational hotspot. This may change with the introduction of NGS techniques: recently, the presence of a mutation was found in 25.9% of 814 patients, and its residual level at the end of therapy linked to different prognosis by using median mutation burden as cut-off [59].

(4) The MLL-MLLT3 fusion gene, determined by t(9;11)(p22;q23) is very rare in adult AML patients, accounting for 2% overall, whereas is more common in children (>10%). It has nonetheless been studied as an MRD marker by RT-qPCR, with low relapse rate (11%) and good 2-year OS (70%) in patients achieving MRD negativity, whereas all MRD+ patients experienced relapse and died [60].

## 4. Multiparameter Flow Cytometry

Another approach to MRD is by multiparameter flow cytometry (MPFC). AML blasts present a distinct immunophenotypical pattern of lineage-specific antigen expression that is not detectable in healthy BM cells [61,62]. Although possessing a slightly less powerful sensitivity, ranging between 1 × 10^−3^ and 1 × 10^−5^ BM cells [18], MPFC has several advantages over molecular biology that make it a complementary rather than alternative technique. Its use for MRD implies the identification of a stable leukemia-associated aberrant immunophenotype (LAIP) [61], detectable at diagnosis in approximately 85% of AML [18]. Alternatively, residual leukemic cells may be evaluated by detecting “different-from-normal” cells, by serial analysis by preloaded tubes for each major committed lineage of healthy BM assessed at time of recovery from myelotoxic chemotherapy [5,6]. The first approach requires to identify at diagnosis one or more leukemic subclones with antigen aberrations, such as the expression of antigens not normally expressed by myeloid cells during commitment (e.g., CD7, CD19, CD56), or the co-expression of markers normally expressed at different, not overlapped stages of maturation, or quantitative over- or under-expression of markers of myeloid commitment (e.g., CD33, HLA-DR). The second approach identifies leukemic cells mainly by subtracting them from healthy BM progenitors, and was developed in the setting of pediatric patients to overcome the possibility of immunophenotypical shifts in AML cells from diagnosis to relapse, and the frequent absence of a proper LAIP analysis performed prior to chemotherapy [5,62].

Most centers focus on LAIP detection; despite this, the technique still has some drawbacks, concerning its sensitivity, which depends on the percentage of blasts presenting with that LAIP at diagnosis, specificity, which is influenced by the degree of aberrancy as compared to background [63,64,65] and LAIP stability over time [66].

MPFC can be used in the context of MRD in at least three different ways:

(1) As an instrument to measure submicroscopic MRD.

In a large HOVON-SAKK study on younger patients, MRD status either post-induction or post-consolidation therapy impacted relapse rate, Relapse-free Survival (RFS) and OS, and proved an independent prognostic factor at the multivariate analysis either in the whole group and in the group at intermediate risk [67]. Cut-off was 0.1% of WBC in PB. Similar data have been reported also on older patients treated within the UK-NCRI trials: MRD negativity conferred superior RFS either when achieved after the first cycle of chemotherapy (i.e., induction) (3-year RFS 42% vs. 26%, *p* = 0.001) or the second one (3-year RFS 38% vs. 18%, *p* < 0.001) [68].

The group of Rome has performed extensive research on MRD measured by MPFC and LAIP detection methods; in all published studies, post-consolidation MRD persistence was adversely associated with CIR and survival, with cut-offs 3.5 × 10^−4^ for BM and 1.5 × 10^−4^ for PB [69,70]. MRD persistence retained strong prognostic value also in patients undergoing autologous HSCT, with CIR of 100% vs. 26.3% in MRD+ vs. MRD− pts (*p =* 0.00004) [71] and 89% vs. 26% (*p* < 0.001) [72] in two consecutive studies.

MPFC MRD determination may also be successfully integrated with baseline cytogenetic characteristics, as done by Buccisano et al. [73,74]: patients with favourable and intermediate cytogenetics who achieved MRD negativity had 4-year RFS of 70% and 63%, and 4-year OS of 84% and 67%, respectively (*p* < 0.001 for all comparisons); those with favourable and intermediate cytogenetics, but with detectable MRD, experienced 4-year RFS of 15% and 17%, and OS of 38% and 23%, respectively (*p* < 0.001 for all comparisons). MRD predictive power was conserved in FLT3-ITD+ patients (4-year RFS 54% vs. 17%, *p* < 0.0001). Therefore, in their analysis MRD proved a more powerful predictor of prognosis than baseline cytogenetic/molecular assessment [18].

(2) To identify the persistence of leukemia-initiating cells (LI-C).

According to a consolidated theory, AML is composed of a hierarchically organized population of cells derived from the clonal progeny of an original transformed HSC, but rapidly evolving as subpopulations defined by additional stochastic mutations and competing one another for dominance [17,75]. After chemotherapy, relapse is determined by the persistence of chemo-refractory, quiescent leukemia-inducing cells (LI-C), also referred to as “leukemia stem cells” (LSC), originally identified retrospectively by means of serial transplantation in immunodeficient mice in a lineage-negative/CD34+/CD38− compartment [75,76,77,78]. It could be argued that MRD evaluation overlaps with the identification of LI-C following therapy [7,79]. Also, identification of LI-C/LSC could complement other MRD tests by selecting the clone ultimately responsible for relapse from those, either characterized by age-related or preleukemic mutations, unable to cause leukemia recurrence even though expressing the MRD marker [7]. By MPFC, LI-C/LSC are now considered CD34+, CD38−, CD123+, CD96+, CD47dim, CD90−, CLL-1+, TIM3+, ALDH-1+, CD99+ [80,81,82]. It has been shown that patients presenting higher proportion of LSC (in this study defined as CLL-1+/CD34+/CD38−) demonstrate significantly lower RFS than patients with less LSC [83]. By combining LSC detection with MRD persistence four groups were identified, with LSC-/MRD− group presenting the best prognosis and LSC+/MRD+ the worst [78]. Recently, a single 8-color tube has been designed to detect LI-C/LSC, based on CD45, CD34, CD38, CD45RA, CD123, CD33, CD44 and a cocktail of multiple markers in a separate fluorescence channel (CLL-1/TIM-3/CD7/CD11b/CD22/CD56) [84].

(3) As a tool to measure leukemia chemosensitivity based on early blast disappearance.

The German AML cooperative group pioneered the use of MPFC to evaluate early debulking, and thus chemosensitivity of leukemic blasts, by MPFC performed early on after initial intensive chemotherapy; they found that MRD persistence at day 16 and the entity of decrease between day 16 and day 1 independently predicted prognosis in terms of CR rates, EFS and OS [85,86]. Other studies later confirmed the prognostic power of early MRD clearance (at day 14), using PB as well as BM [87,88,89], in the group at intermediate-risk patients (5-year RFS 15% vs. 37% in MRD+ vs. MRD−, *p =*.016), normal-karyotype/NPM1-mutated patients (5-year RFS 13% vs. 49%, *p* = 0.02) and in FLT3-ITD+ patients (3-year RFS 9% vs. 44%, *p* = 0.016) [89]. MRD disappearance at these very early time-points was also positively correlated with the achievement of CR and the quality of hematopoietic recovery [90].

Despite its advantages, MRD detection by MPFC presents some drawbacks, such as the lack of inter-lab standardization in antibody panels and instrument configuration and the need of a specific training. Difficulties in collecting enough events after cell sorting, possible shifts of LAIP from diagnosis to relapse, and the absence of adequate LAIP at diagnosis also limit the applicability of MPFC for MRD assessment.

It is therefore logical that approaches combining molecular techniques and MPFC have been tested and had good results.

## 5. Combined Approaches 

Due to its widespread applicability, WT1 has been extensively studied in combination with MPFC. Marani et al. [91] demonstrated lower DFS in patients with MPFC MRD after induction >1 × 10^−2^ (79.5% vs. 27.3%; *p* = 0.032) or with WT1 reduction <1.5 log (46.2% vs. 0%, *p =* 0.001). Rossi et al. [92] demonstrated that post-induction reduction of WT1 lower than 1.96 log and post-consolidation MPFC MRD >0.2% were significantly related to CIR and DFS. In a follow-up article, the same authors [93] identified MPFC and WT1 MRD persistence as a predictor of relapse and DFS at all pre-transplant checkpoints and at 1-month post-allo-HSCT. Furthermore, they found that post-consolidation BM WT1 persistence (82% vs. 51%, HR: 4.1, *p* = 0.02) and MPFC MRD positivity (81% vs. 57%, HR: 3.3, *p* = 0.0001) predicted worse RFS [93].

Along studies using a combined approach at MRD, we also briefly report the results of our own [94]. Among 151 AML patients, 126 achieved CR (83.4%), and 54 pts relapsed after median time 8 months, with an overall 1-year CIR of 30%. WT1 was overexpressed at diagnosis in 78% of patients; 23% of all patients remained WT1+ after induction and 12.6% remained WT1+ after consolidation. A higher proportion of MRD+ patients was detected at the same time-points using MPFC (57% and 43.6%, respectively). Univariate analysis showed that any MRD positivity, determined either by WT1 or MPFC MRD, predicted a significantly higher 1-year CIR: 59.2% and 66.7% of patients who resulted WT1 MRD+ post-induction and consolidation relapsed, respectively, compared to 35.4% and 40.2% of MPFC MRD+ patients at the same time-points. By combining WT1 and MPFC assays, we identified 3 groups: (1) MRD double-positive patients (including WT1 MRD+ lacking MPFC evaluation); (2) discordant MRD patients (including patients with MPFC MRD+ lacking WT1 evaluation); (3) concordant MRD double-negative patients. At the post-induction time-point, 1-year CIR ranged from 60% in group 1 to 6.5% in group-3; at the post-consolidation time-point, group-1 patients had a 1-year 85% CIR vs. 12% in group-3. When assessed post-consolidation, this score reliably predicted 1-year CIR also after allo-HSCT.

Besides WT1, very little has been published on the use of combined MPFC and RT-qPCR for MRD assessment using other molecular markers. As far as RUNX1/RUNX1T1 and CBFB/MYH11 are concerned, Ouyang et al. [95] found no agreement on 93 patients overall between RT-qPCR and MPFC results when evaluated post-induction (*k* coefficient test = 0.041), and only weak agreement during consolidation (*k* = 0.083), follow-up (*k* = 0.164) and rescue therapy (*k* = 0.376). Despite this, MPFC proved of some value in discriminating groups at different relapse risk in those patients with post-induction RT-qPCR MRD at intermediate levels (i.e., in the 0.1–1% and 1–10% range) [95]. Furthermore, NPM1 is part of several multigene NGS MRD panels currently studied by ongoing trials. We believe further research is needed before drawing conclusive evidence on this topic.

## 6. Issues in the Implementation of MRD Assessment as the New Standard to Evaluate Clinical Response

MRD techniques hold the potential to enable physicians to more properly judge the response to therapy in CR patients, to decide whether to switch to alternative, and usually more toxic, treatments given as consolidation before the disease progresses. As new targeted agents and cell-based therapies become available, MRD could also provide an essential tool for selecting the best candidates for these therapies and for measuring their effects. Outside clinical trials, though, few clinicians will currently opt to administer potentially harmful treatments to patients in CR on the basis of MRD determinations alone. In fact, Jourdan et al. observed that only 12 out of 52 CBF AML patients directed per protocol to allo-HSCT because of poor MRD response actually performed it [29]. Caution has been advocated to use MRD as clinical-decision tool outside controlled clinical trials [79]. This is because of several issues that we address in the following section: 

### 6.1. Inter-Laboratory Standardization

Most studies in the last few years have been conducted on a national-based program, with the shipment of study samples by peripheral centers in few big hub laboratories equipped with the facilities and the expertise to perform MRD evaluation. As such, proper expertise and standardization is still spreading among laboratories not directly involved in the pilot studies [5,79]. This includes pre-analytical workflow, threshold definitions and reporting guidelines that have not been internationally defined yet for AML, as done, for instance, for chronic myeloid leukemia [7].

### 6.2. Choice of Methodology

It is currently unknown which MRD evaluation method, if any, is the best for clinical purposes. Some MRD methods, especially MPFC, rely on the technique as much as on personal expertise and convictions of the operator [79]. The distinction between LAIP and normal hematopoietic clones, e.g., in a regenerating BM, may require 8–10 fluorochrome phenotypic analysis, which is cumbersome for many [18]. Immunophenotypical shifts in LAIP, probably because of the outgrowth of chemoresistant subclones with different aberrancies, may hamper the reliability of MPFC MRD evaluation [96]. Centralizing all samples in few hub laboratories might prove unpractical in the long term, due to the need of an efficient network, frequent external quality controls, and may generate excess workload for the hub laboratories involved.

### 6.3. Best Source for MRD and Sample Quality

In fact, sample quality, hampered by hemodilution after repetitive BM sampling from the same point, as well as by deterioration during transport, may become a crucial point in MRD evaluation [7,79]. The use of PB reduces MRD sensitivity as compared to BM by an approximately estimated 1–1.5 log [6,79]. For NPM1, a 1.0 log reduction in sensitivity has been reported in some studies [39,40], while others defined a reduction of sensitivity ranging from 0.6 at diagnosis to 1.1 log at follow-up [43]. Exact measurements of the difference in MRD assay due to sample source depend on the marker used for MRD assessment, on the time-point used for evaluation, as well as on other methodological variables. Despite inferior sensitivity, though, Zeijlemaker et al. recently confirmed in flow cytometric data a good correlation between the findings in PB and BM, and even reported a better predictability of PB results as compared to BM [97]. For some markers, the difference in sensitivity might become less relevant for the final interpretation of results at later time-points; for instance, WT1 was reported as equally or even more reliable when assessed in PB than BM during the follow-up of CR patients, because of the lack of background expression by non-leukemic BM cells [47]. Similarly, analysis of CBFB/MYH11 in paired BM and PB samples revealed higher sensitivity of BM during treatment, whether in follow-up PB proved equally informative, and could be obtained with less invasive sampling [26]. Finally, the fraction of NPM1-negative PB samples still showing detectable MRD in the BM decreased from 46% to 18% from the induction and consolidation periods to follow-up monitoring [43]. BM might not be the optimal source for MRD also because of the heterogeneous involvement of bone spaces by leukemic cells, especially after therapy: studies using 3’-deoxy-3’-[18F]fluoro-1-thymidine positron emitting tomography to evaluate BM response after the first course of chemotherapy [98] showed how response locally varies after chemotherapy throughout the pelvic region. This is in line with previous reports on rats, where homogeneous distribution of AML cells in the skeleton was remarkably lost after therapy [99].

### 6.4. Significance of MRD Evaluation as a Decision-Making Tool

Until recently, most studies on MRD in AML have been designed to evaluate only the prognostic significance of MRD, without changes in therapies determined per protocol by MRD. Thresholds for significance may vary among studies according to the selected time-points, the overall therapeutic program, and the specific dose-intensity of consolidation, especially in elderly patients [12]. As such, conclusive evidence on the merit of consolidation choices based on MRD determination can only be obtained by prospective trials that are mostly ongoing (Table 2).

Still, some considerations may be taken from the experience and the available literature: most studies have demonstrated how the reappearance of MRD during follow-up after previous achievement of MRD negativity is indicative of disease reoccurrence in median 2 months [6,79]. More controversial is the significance of MRD persistence above thresholds after induction or consolidation, where we, among others, believe the trend of transcript reduction to be more indicative of subsequent relapse than absolute values [20,24,27,28,29,39]. Therefore, it is generally advisable to check a positive PCR in new samples obtained with a span of at least a fortnight, to confirm the diagnosis of molecular relapse. Persistence of detectable mutations at stable, very low levels, especially in the case of DNMT3A [39,53] and RUNX1/RUNX1T1 [27], may be compatible with persisting long-term remission.

It is unclear whether all considerations about MRD as decision-making pool apply to elderly (i.e., >60–65-year old) AML patients, an age group where controlled studies are lacking, few therapeutic advancements have been done, long-term OS is still about 5–20% since decades (also in the cases treated with intensive chemotherapy), and for whom allo-HSCT may often not be an option. In the context of the EORTC/GIMEMA trials, Buccisano et al. [100] observed higher rate of MPFC MRD persistence after therapy in elderly (i.e., >60 years old) as compared to younger patients (89% vs. 72%, *p* = 0.009), and attributed this finding to less intensive post-remission therapy. Elderly MRD+ patients later showed very high relapse rate (83%). Interestingly, also those elderly patients that achieved MRD negativity eventually relapsed twofold more than younger MRD-negative patients (42% vs. 24%, *p* = NS), even if statistical significance was not reached. This data suggests that MRD negativity might have different implications for elderly patients. The recent introduction of post-transplant Cyclophosphamide has expanded the possibility to perform haploidentical allo-HSCT also in the setting of elderly patients [101], improving results and the rate of donor availability. We, in our personal opinion, support the use of allo-HSCT in all fit patients up to 70 years of age, should MRD be positive at the end of consolidation in cytogenetically intermediate or adverse-risk patients, and their Hematopoietic Cell Transplant-Comorbidity Index (HCT-CI) score below 3 [102]. However, it is worth pointing out that at the state of the Art this practice is not based on high-quality clinical evidence. Randomized trials would be needed to properly address the advantage of performing allo-HSCT or other forms of MRD-directed therapies in such conditions [7]. Increasing age (>60 years) has proved detrimental for OS also in favourable-risk AML [2,3,23].

### 6.5. Safety and Efficacy of Treatment Alternatives

The whole concept of using MRD to decide between treatment alternatives depends on the risk-benefit ratio of available therapies. At present, a major field in the application of MRD assessment in AML is the choice of best consolidation in intermediate-risk patients achieving CR1. Two major meta-analysis proved the advantage of allo-HSCT in this setting [8,9]. Still, results have been more heterogeneous in other studies [67,103], especially in terms of OS among patients older than 40 years, because of the high TRM (15–25% [10,12]) still hampering allo-HSCT, as well as the profound impact on the quality of life by transplant complications and Graft-vs-Host reactions [12]. At the same time, however, it is possible to envision other practical use of MRD assessment, such as the selection of patients at the end of consolidation therapy still needing additional maintenance with less toxic therapies, such as monoclonal antibodies (e.g., anti-CD33, anti-CD123, BiTe antibodies), hypomethylating agents, or specific kinase inhibitors (e.g., anti-FLT3, anti-KIT). Several studies are currently testing this hypothesis (Table 2).

### 6.6. Effectiveness of allo-HSCT in Eradicating MRD

There is consistent data pointing out at how even allo-HSCT might be limited in eventually curing MRD+ AML patients. Pretransplant MRD status is predictive of relapse and survival in a variety of studies [104,105]. With cut-off of 0.1%, Walter et al. reported a 3-year OS of 73% vs. 32%, and a relapse rate of 21% vs. 58%, in patients transplanted in CR1, depending on their pre-transplant MRD status (*p* < 0.0001) [104]. These results were updated in 359 transplanted adults, with 3-year relapse rates of 22% vs. 67% for MRD− vs. MRD+ patients, resulting in an OS of 73% vs. 26% [106]. This was confirmed by another study that included both myeloablative (3-year CIR 22% vs. 63%) and non-myeloablative conditioning (3-year CIR 28% vs. 57%, *p* < 0.0001) [105]. The time-point at 28 ± 7 days from allo-HSCT was shown as predictive for survival [107]. These results are comparable to what reported for patients allotransplanted with morphologically evident disease [6,10,12]. One way to improve this might involve changes in conditioning regimens or MRD-directed post-allo-HSCT maintenance therapy. 

### 6.7. Best Time-Points for MRD Evaluation 

Besides regular monitoring at each block of chemotherapy preceding allo-HSCT, the optimal schedule during follow-up or after allo-HSCT remains unclear, and may depend on leukemia subtype. A mathematical model has been proposed based on the kinetics between molecular and clinical relapse [108]: patients with leukemias characterized by slow relapsing pattern, such as CBFB/MYH11 AML, would need one MRD determination in PB every 6 months to detect 90% of relapses at a median preceding time of 60 days; conversely, BM testing at more frequent intervals would be necessary in other subtypes, such as those characterized by RUNX1/RUNXT1 or NPM1 mutations, with or without FLT3-ITD [108]. Similarly, Hokland et al. proposed a model by which RUNX1/RUNX1T1 and CBFB/MYH11, on one hand, present a molecular relapse that usually precedes the hematological relapse up to 1 year [20,50], while WT1 molecular relapse, on the other, usually briefly precedes (by 1–2 months) clinical recurrence [50], and mNPM1 AML widely varies in relapse kinetics, depending on concomitant FLT3-status (with shorter time frame in FLT3-ITD+ patients) [41]. Based on these data, guidelines for MRD follow-up have been proposed [45]. Adherence to these guidelines statistically allows the detection of 85% of relapses, with a minimal time frame of 60 days [50]. Our approach follows the guidelines by Ommen et al. [45]. After allo-HSCT we measure MRD monthly during the first 3 months, and then every 3 months for the first 24 months; additional measurements are performed in the case of positive MRD findings or incomplete donor chimerism. 

## 7. “Real-Life” Use of MRD as a Decision-Making Tool in 2017

As a rule of thumb, when considering MRD assessment outside a clinical trial, we would like to stress the importance of applying the same rigorous methodology that was tested in the clinical trials. This means to measure MRD at fixed time-points with more than a single technique, when possible, and to base final decisions on the evaluation of results obtained by the same method of MRD assessment throughout the clinical history of the patient, considering the overall trend, besides single measurements at specific time-points [7]. Even so, as we previously argued, several issues still hamper the widespread diffusion of MRD techniques as tools for treatment decisions outside clinical trials. Among these, the most important is the need for additional inter-laboratory standardization of the analytical techniques, especially in the case of MPFC and NGS. As such, a word of caution is, in our opinion, still needed when using MRD results outside a clinical trial to decide for treatments potentially responsible of higher morbidity and mortality; this is typically the case of the choice of allo-HSCT as consolidation for cytogenetically favourable-risk patients with slowly-achieved morphological CR, and for intermediate-risk AML patients in CR1, a setting where the rate of treatment failure still approaches 40–50%. For intermediate-risk patients, we believe that the prognostic power of MRD has already been extensively demonstrated, especially when MRD be performed by combined molecular (RT-qPCR) and MPFC techniques. Therefore, we would consider MRD persistence at the end of consolidation, or MRD recurrence after initial clearance, as the most significant risk factor to take into consideration together with baseline risk assessment and transplant risk (e.g., by HCT-CI [102]) to reach a final decision. We would not, however, automatically switch MRD+ intermediate patients to allo-HSCT without a proper consideration of the other prognostic factors (such as, e.g., high WBC count at diagnosis), of the performance status of the patient, his infectious history and transplant risk. Rather than substituting baseline evaluation, MRD evaluation should in our opinion complement that, as a unique instrument to evaluate residual disease burden and overall disease chemosensitivity. This latter depends on several issues, such as drug schedules, dose-intensity, biodistribution, and the pharmakogenetic profile of the patient, that cannot otherwise be reliably assessed on an individual basis. Similarly, we would suggest to consider MRD persistence after consolidation in cytogenetically favourable AML, such as CBF AML or normal-karyotype wtFLT3 mNPM1 patients, as a very strong indicator for consolidation with allo-HSCT in CR1, but only after a proper assessment of patient status and transplant-risk. In both favourable- and intermediate-risk patients, given the current level of uncertainty on the efficacy of allo-HSCT in eradicating leukemia from MRD+ patients, we believe alternative treatments should be offered, even if results are poorer, and the final choice whether or not to undergo allo-HSCT in CR1 be shared with the patients themselves. Should experimental trials with immunotherapy or maintenance therapy be available, we would offer them to MRD+ patients. Finally, in case of no better option, we would institute closer surveillance during follow-up.

MRD reappearance or persistence after allo-HSCT should especially be taken into account: in our opinion, this constitutes an important alarm prompting for preemptive intervention, such as faster withdrawal of immunosuppression or implementation of Donor Lymphocyte Infusions. Should experimental trials be given, hypomethylating therapy or treatment with specific kinase inhibitors would be valid alternatives.

**Figure 1:**
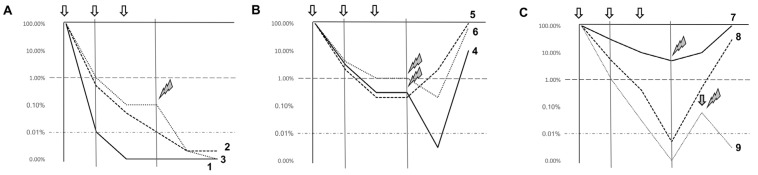
Paradigmatic scenarios of AML evolution after therapy considering MRD results. Nine paradigmatic scenarios (Cases 1 to 9) are displayed. Trends are simplified as compared to real life, for explicative reasons. Intermittent line at 1% represent the usual sensitivity limit of morphological examination; dotted line at 0.01% represents the usual sensitivity limit of MRD assessment. Vertical arrows represent chemotherapy cycles. Vertical lines represent common time-points for evaluation, i.e., after induction therapy and consolidation. (PANEL **A**): in Case 1, a quick response to chemotherapy cycles (white arrows) allows the patients to reach morphological CR after induction and the disappearance of measurable MRD after the first consolidation cycle. MRD remains undetectable during follow-up and the patient stays long-term in clinical remission. In Case 2, MRD negativity is reached within the end of consolidation therapy, so no further treatment (e.g., with allo-HSCT) is decided, and the patient remains in stable MRD negativity and clinical remission in the long term thereafter. In Case 3 the patient retains MRD-detectable disease at the end of consolidation, and allo-HSCT (lightning bolt) is decided; this enables us to obtain MRD negativity and long-term remission. (PANEL **B**): in Cases 4, 5 and 6 morphological remission is obtained following induction therapy, but no MRD is obtained by the end of consolidation in either cases. Case 4 is then consolidated by allo-HSCT, which enables the patient to reach MRD-negative leukemia levels briefly, but ultimately fails to eradicate the disease, with eventual relapse. In Case 5, no allo-HSCT is performed, and leukemia rapidly evolves from MRD persistence into overt clinical relapse. In Case 6, MRD identifies leukemia persistence just below the level of morphological detection (1%), and allo-HSCT, though inducing a brief reduction of residual disease, does not manage to obtain MRD negativity nor to prevent ultimate relapse. (PANEL **C**): in the scenario represented by Case 7 the patient never achieves a proper control over the disease, which results primary refractory; allo-HSCT is used with little efficacy, and ultimately clinical progression is unavoidable. Finally, Case 8 and 9 experience deep early response, achieving MRD negativity within the end of consolidation. In both cases reappearance of AML is detected by MRD monitoring during follow-up before clinical relapse: Case 9 is treated with additional therapy (either experimental treatments—i.e., grey arrow—or, less likely, allo-HSCT, lightning bolts) and restored to MRD negativity. Case 8 does not receive such treatment and ultimately relapse. In Case 9 the possibility of a molecular relapse spontaneously reverting to MRD-negativity also without the need for a clinical intervention, although increasingly rare with modern MRD measurement technique, cannot be ruled out, especially in the case of CBF AML.

In Figure 1 we summarize possible scenarios in which MRD assessment could affect patient management in “real-life” practice.

## 8. Some Final Considerations about Future Developments

MRD evaluation is becoming the new standard in evaluating response in AML [5,6,79]. In the near future, inter-laboratory standardization of the analytical techniques will probably be improved by the building of functional networks for referral hub laboratories, as currently done by the GIMEMA AML Labnet group. Technical recommendations for MRD detection and its clinical use by the European LeukemiaNet are also expected shortly.

The NGS methodology holds the promise of improving our knowledge on the pathogenesis of leukemia, through the concomitant evaluation of multiple molecular new markers (such as IDH1/2 [55], RUNX1 [42,43], TET2 [6]), together with established ones, such as NPM1 [42,43], and through the possibility of serially analyzing the clonal evolution of AML [16,17].

Another field of significant development in the near future will be the validation on a large scale of MPFC-based methods to reliably quantify residual LI-C/LSC [77,84], which might lead to specific therapies aimed at eradicating LI-C/LSC.

MRD persistence after allo-HSCT should also become an intense field of investigation, prompting the use of novel, well-tolerated agents. Finally, MRD determination could also soon become a surrogate end-point for survival, thus enabling faster clinical approval of novel drugs [7].

Despite these advances, individual risk assessment and personalization of treatment will always require the global evaluation of the disease and the person suffering from it, with their unique clinical history. As such, even when all MRD final technical issues are resolved, a comprehensive medical evaluation, together with the sensitivity of a human mind, will still be needed in the future.

## Figures and Tables

**Table 1:** Potential molecular markers for Minimal Residual Disease in Acute Myeloid Leukemia (excluding Acute Promyelocytic Leukemia).

Molecular Markers	Frequency (% of All)	Occurrence in Leukemogenesis	Predictive Power for Clinical Relapse	Technique
*Fusion products*				
RUNX1/RUNX1T1	7–10%	Early	Very good	RT-qPCR
CBFB/MYH11	5–8%	Early	Very good	RT-qPCR
MLL/MLLT3	2%	Probably late	Good	RT-qPCR
*Mutations*				
FLT3-ITD	25–30%	Late	Poor	RT-qPCR/NGS
NPM1	30% (50% in normal-karyotype)	Late	Very good	RT-qPCR/NGS
DNMT3A	10–15%	Early	Poor	NGS
RUNX1	10%	Early	Possibly good	NGS
IDH1/IDH2	8–9% each	Early	Possibly good	NGS
*Overexpression*				
WT1	85–90%	Unknown	Good	RT-qPCR

Note: RUNX1/RUNX1T1, runt-related transcription factor 1/runt–related transcription factor 1 translocated to 1; CBFB/MYH11, core-binding factor subunit beta/myosin heavy chain 11; MLL/MLLT3, mixed-lineage leukemia; NPM1, mutated nucleophosmin1; FLT3-ITD, Fms-like tyrosine kinase Internal Tandem Duplication; MLL-PTD, mixed-lineage leukemia Partial Tandem Duplications; WT1, Wilms’ Tumor gene.

**Table 2:** Major ongoing clinical trials on Minimal Residual Disease in Acute Myeloid Leukemia in adults.

Trial	Nation	ID	MRD-Related Endpoints	Type	Age Limits
**MRC *AML 17***	UK	ISRCTN55675535	Assess the prognostic value of minimal residual disease monitoring (randomization: monitoring vs. not monitoring)	Phase 3	<60 years
**MRC *AML 19***	UK	ISRCTN31682779	Assess the prognostic value of minimal residual disease monitoring (randomization: monitoring vs. not monitoring)	Phase 3	18–60 years
**MRC *AML 18***	UK	ISRCTN78449203	Treatment intensification in MRD+ patients after the first cycle, chemotherapy randomization	Phase 3	>60 years
**GIMEMA *AML1310***	Italy	NCT01452646	MRD stratification of intermediate-risk karyotype; risk-adapted, MRD-directed therapy (autoSCT vs. SCT) after first consolidation	Phase 2	18–60 years
**CETLAM *AML-03***	Spain	NCT01723657	MRD stratification of intermediate-risk karyotype; risk-adapted, MRD-directed therapy (autoSCT vs. SCT) after first consolidation	Phase 2	18–70 years
**PETHEMA *LMA10***	Spain	NCT01296178	Risk-adapted, MRD-directed therapy(study arms not provided)	Phase 3	<65 years
**PETHEMA**	Spain	NCT00390715	Prospective study on the prognostic value of baseline cytogenetics and MRD monitoring	Observational (prospective)	<65 years
**Nanfang Hospital of Southern Medical University, Guangzhou**	China	NCT02870777	MRD-directed therapy for low- and intermediate-risk AML. Front-line allo-HSCT intensification is programmed for MRD+ patients	Phase 3	18–60 years
**Rochester University**	USA	NCT01311258	Identification by MPFC, among all MRD cells, of the clones eventually responsible for clinical relapse (LIC)	Observational (prospective)	>18 years
**Az. Ospedaliera Città della Salute e della Scienza Torino**	Italy	NCT02714790	Assess the prognostic role of MRD defined as BM expression of WT1	Observational (retrospective)	>18 years
**Medical College of Wisconsin**	USA	NCT02349178	Estimating the efficacy of Clofarabine, Cyclophosphamide and Etoposide in eliminating MRD in AML patients, otherwise in clinical remission, before allo-HSCT	Phase 2	<40 years
**Technische Universitat of Dresden RELAZA2**	Germany	EudraCT 2010-022388-37	5-Azacitidinetreatment of patients with MDS or AML with significant residual disease or an increase of MRD	Phase 2	>18 years
**Ulm University**	Germany	NCT01770158	Maintenance Therapy with Histamine Dihydrochloride and Interleukin-2 in AML MRD+ patients post consolidation therapy	Observational (prospective)	>18 years
**Washington University**	USA	NCT00863434	Clofarabine and Cytarabine in treating MRD+ (by MPFC) AML patients	Phase 2	18–75 years
**Singapore General Hospital**	Singapore	NCT00394381	Autologous Cytokine-induced Killer cell adoptive immunotherapy for MRD+ patients post autologous HSCT	Phase 1/2	12–75 years
**Institute of Hematology & BloodDisease** **Hospital, Tianjin**	China	NCT03021395	Efficacy of maintenance Decitabine (after consolidation chemotherapy) in clearing MRD in patients in clinical remission	Phase 1/2	14–55 years
